# Previous exposure to an automated milking system before parturition is associated with performance of first-lactation cows on Canadian dairy farms

**DOI:** 10.3168/jdsc.2026-1121

**Published:** 2026-09-07

**Authors:** J.E. Brasier, R. Bergeron, D.B. Haley, T.J. DeVries

**Affiliations:** 1 Department of Animal Biosciences, University of Guelph, Guelph, ON, N1G 2W1, Canada; 2 Department of Population Medicine, University of Guelph, Guelph, ON, N1G 2W1, Canada

## Abstract

The objective of this study was to determine if previous exposure to an automated milking system (AMS) before first calving is associated with the performance of first-lactation cows when milked on an AMS on Canadian dairy farms. A matched pair study design was used. Initially, 197 Canadian AMS farms were enrolled in 2019, where AMS data (with dates ranging from January 2018 to December 2020) was available to be collected from 121 farms using Lely AMS. Of the 121 farms, those that were majority Holstein, had a free-flow traffic system, and housed cows in a freestall barn were considered (n = 102). Farms were categorized as herds indicating in the survey that they exposed first-lactation cows to the AMS before the first calving (PRE; n = 27), or as herds indicating in the survey that first-lactation cows had no previous exposure to the AMS before the first milking on the AMS (NPE; n = 75). Farms were pair matched (1 farm per training category) based on region, bedding type, the major forage type in their ration, and if they built a new barn when converting to AMS, for a total of 27 pairs. Data from the first-lactation cows on these farms were averaged by farm and DIM (3–305), then analyzed at the herd level. From 3 to 30 DIM, first-lactation cows on PRE farms had greater milking frequency and a shorter milking interval compared with cows on NPE farms. The first-lactation cows on PRE farms had either greater milk yield and AMS concentrate delivered, or tended to have greater milk yield and AMS concentrate delivered, at different time points from 3 to 305 DIM, and had a lesser SCC linear score from 3 to 200 DIM. Finally, the first-lactation cows on PRE farms also had lower odds of a milking failure from 3 to 60 DIM and from 121 to 200 DIM compared with those on NPE farms. Overall, providing first-lactation cows with previous exposure to the AMS before first calving was associated with improved adaptation during the first 30 DIM and improved production across the lactation compared with farms that did not provide previous exposure.

## Graphical Abstract

**Figure:**
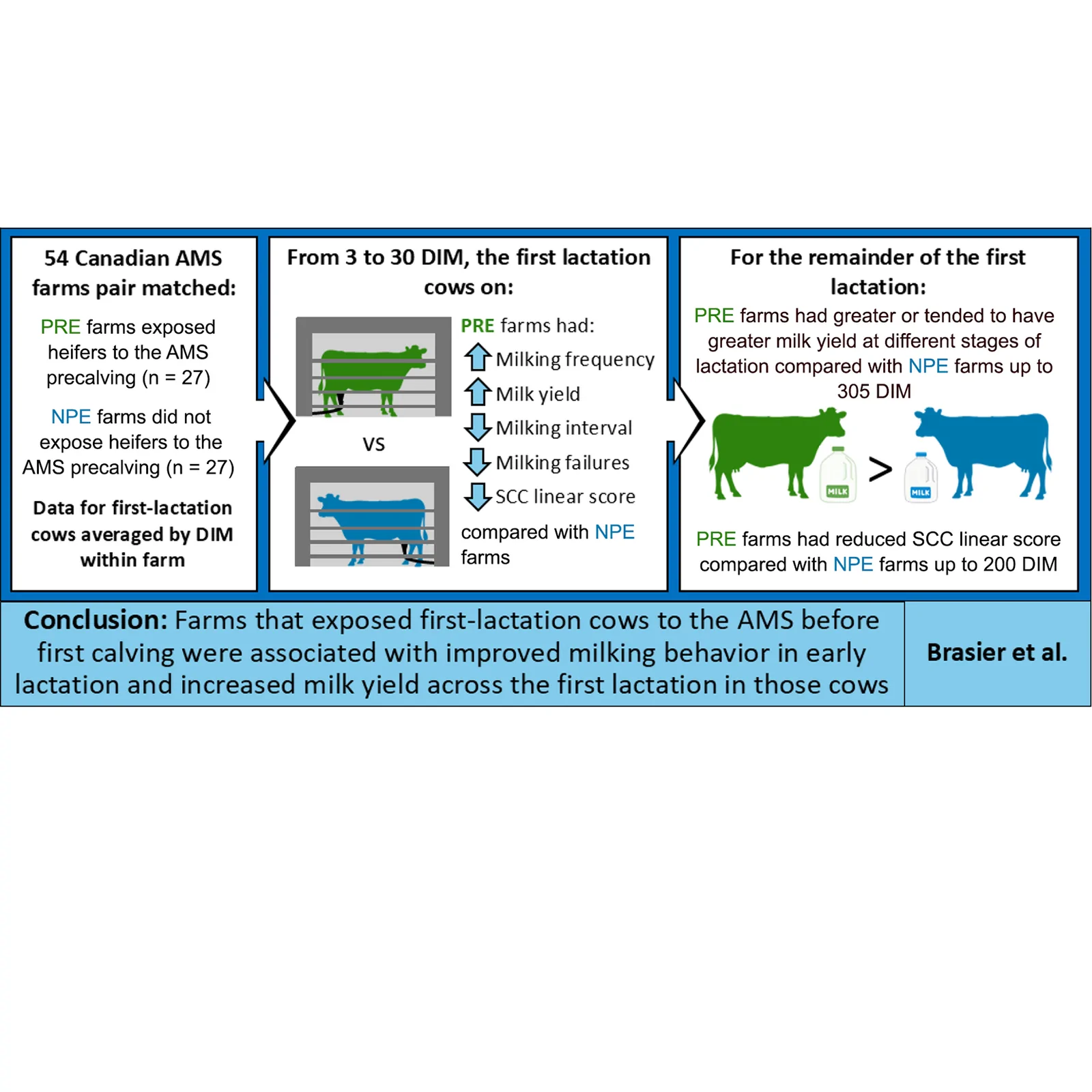

A foundation of successful automated milking system (**AMS**) management is ensuring voluntary milkings, where cows visit the AMS on their own to be milked without human intervention (Tse et al., 2018; Brasier et al., 2025a). Cows who do not voluntarily visit must be fetched by the producer, which increases labor. Voluntary milkings, visits, and fetching events can, thus, be used as measures of adaptation to the AMS (Brasier et al., 2025a). Multiple factors influence how well cows adapts to AMS, including parity and previous exposure to the AMS (Jago and Kerrisk, 2011).

A growing body of evidence suggests that previous exposure, or training, to the AMS before first milking can improve adaptation and increase production when milking on the AMS. Previous exposure can either be passive or active (Brasier et al., 2026a). Passive training involves letting cows voluntarily explore or enter AMS, without instructing cows to enter. This can include using a phantom AMS (von Kuhlberg et al., 2021) or giving cows time in an AMS pen before first milking, in order to acclimate to the pen as well as observe and explore the AMS (Brasier et al., 2026b). Passive training can reduce fetching events (von Kuhlberg et al., 2021; Brasier et al., 2026b) and improve milk yield (Brasier et al., 2026b). Alternatively, active training involves instructing cows to enter the AMS, following a strict training protocol. More studies have been done on active training, with documented benefits in terms of greater voluntary visits and milk yield across at least the first few weeks of AMS milking (Widegren, 2014; Whitehead, 2024; Brasier et al., 2025a,b).

Limited data are available on how training is being used on commercial farms, as well as on the effects of that training. In a study of 38 AMS farms in the Upper Midwest of the United States, only 9% of respondents trained first-lactation cows before first calving (Swartz, 2022). No effect of training on production or behavior was detected in that study, likely due to the low number of farms that trained first-lactation cows before calving (Swartz, 2022). In a survey completed in Canada, 18% of respondents trained first-lactation cows before first calving, with no comparison of performance between farms (Tse et al., 2018). Further, the long-term effects of training on performance have not been fully explored, outside of a few studies where cows were followed into mid-lactation (Widegren, 2014; von Kuhlberg et al., 2021; Whitehead, 2024). Thus, the objective of this study was to determine if previous exposure to the AMS before first calving is associated with the performance of first-lactation cows when milked on an AMS on Canadian dairy farms, both in the long and short term. It was predicted that farms that introduce first-lactation cows to the AMS before first calving would have improved first-lactation cow performance compared with farms that do not introduce their first-lactation cows to the AMS before calving.

Data for this study were collected as part of a larger study by Matson et al. (2021), whereby the herd-level housing and management strategies of 197 AMS farms from across Canada were benchmarked and associated with milk production and quality. The distribution of farms was representative of the population and distribution of Canadian AMS herds at that time. Consent for data collection was obtained from the producers before visiting the farms between April and September 2019. A survey on housing and management, outlined by Matson et al. (2021), was conducted during the farm visits. Data on nutritional management were also collected, as described by Van Soest et al. (2024). Survey data from Matson et al. (2021) and Van Soest et al. (2024) were used in the current study.

Only herds that were using Lely AMS (Maassluis, the Netherlands) were considered for the current study, as those AMS data were the most complete out of all the systems observed in the Matson et al. (2021) study. Using data from only one AMS system also provided greater parameter consistency. Detailed individual-cow AMS milking records were obtained for 121 Lely AMS herds. Of those 121 herds, herds that were majority Holstein (>90%), had a free-flow traffic system, and housed cows in a freestall barn were considered (n = 102). Of those 102 herds, 27 herds were identified as having indicated in the management survey that they exposed their first-lactation cows to the AMS before their first calving. These farms were categorized as **PRE** farms. The remaining 75 herds indicated that they did not expose their first-lactation cows to the AMS before their first calving and were categorized as **NPE** farms.

Due to the uneven distribution in farms that provided previous exposure to the AMS, as well as the large variation in management practices across farms, farms were pair matched to limit the variation between the 2 training categories (1 per training category; 27 pairs total). The pair matching was done based on region (Western Canada, Ontario, Québec), bedding type (organic vs. inorganic), the major forage type used in their lactating cow diet (corn silage, barley silage, haylage, other), and if they built a new barn when transitioning to the AMS (yes or no; Table 1). These matching criteria variables were chosen based on previous findings: Matson et al. (2021) demonstrated that bedding type was associated with milk yield, whereas Van Soest et al. (2024) demonstrated that region and major forage type in the diet were associated with milk yield. Finally, Swartz (2022) demonstrated that building a new barn when converting to AMS was positively associated with milk yield. Other housing and management characteristics from the survey that were not used for pair matching were used as covariates to account for differences between farms; these are reported by training category in Table 1.

**Table 1:** Descriptive statistics of automated milking system (AMS) farms in Canada that did not give first-lactation cows previous exposure to the AMS before calving (NPE; n = 27 farms) or did provide first-lactation cows with previous exposure to the AMS before calving (PRE; n = 27 farms)

Variable	NPE category	PRE category
Region^1^, ^2^		
Ontario	16 (59)	16 (59)
Québec	6 (22)	6 (22)
West	5 (19)	5 (19)
Bedding type^1^, ^2^		
Organic	17 (63)	17 (63)
Inorganic	10 (37)	10 (37)
Major forage in diet^1^, ^2^		
Corn	16 (59)	16 (59)
Barley	1 (4)	1 (4)
Haylage	4 (15)	4 (15)
Other	6 (22)	6 (22)
New barn built when converting to AMS^1^, ^2^	20 (74)	20 (74)
Tiestall system before converting to AMS^2^	17 (63)	20 (74)
Access to outdoors^2^	2 (7)	4 (15)
Stalls raked^2^ (no./d)		
Once	22 (81)	17 (63)
Twice or more	5 (19)	10 (37)
Feed delivery^2^ (no./d)		
Once	9 (33)	10 (37)
Twice or more	18 (67)	17 (63)
Ventilation system^2^		
Natural only	1 (4)	3 (11)
Combination of natural and fans	22 (81)	18 (67)
Tunnel	4 (15)	6 (22)
Bedding added to freestalls^2^		
Daily	5 (19)	6 (22)
Weekly	18 (67)	17 (63)
Monthly	4 (15)	4 (15)
Herd size^3^ (milking cows/farm)	136.7 (45–580)	94.4 (39–340)
AMS stocking density^3^ (cows/AMS)	50.8 (35–61)	44.6 (30–65)
Years since AMS installation^3^ (yr)	5.1 (1–16)	4.5 (1–15)
Alley cleaning^3^ (no./d)	12.6 (2–40)	11.3 (1–32)
Feed bunk space^3^ (cm/cow)	59.3 (24–122)	63.2 (34–106)
Feed push-ups^3^ (no./d)	15.2 (0–24)	15.4 (0–40)

1 Used to pair match farms.

2 Number of herds in that category, with percentage in parentheses.

3 Average value for herds in that category, with range in parentheses.

Data for milking activity and yield from January 2018 to December 2020 were extracted from the software for the Lely AMS (Time for Cows, Lely Industries N.V.) through the use of TeamViewer (TeamViewer, Goppingen, Germany). An average of 19.5 mo of data were extracted per farm (range: 9.5 to 24.8 mo). Information included the date, time, duration, and the outcome of each AMS visit: successful milking, defined as the cow entering the AMS and being milked out before leaving; milking failure, defined as a cow is milked in the AMS, but was not milked out before leaving; or refusal, defined as a cow visiting the AMS outside of her allowed milking permission, so she left the AMS without milking. Milk yield and SCC, if available, were also collected for each cow on each farm. Four farms from the PRE category and 5 farms from the NPE category did not have SCC data. The AMS measured SCC for each milking with an integrated milk quality control cell count sensor (Lely Industries N.V.), as validated by Lusis et al. (2019). Values for failures, milking frequency, refusals or rejections, visits, milk yield, and concentrate delivered were summed by day for each cow (0000 h to 2359 h). Visit duration per milking (or box time) and milking interval were averaged across each day for each cow, whereas a weighted average of SCC was calculated for each day for each cow. Linear score was then calculated to normalize the SCC data for statistical analyses using the following formula: log_2_(SCC/100,000) + 3 (Wiggans and Shook, 1987).

The AMS settings for both milking permission and concentrate allocation were not able to be obtained, as discussed by Van Soest et al. (2024). Individual cow parity and DIM data were obtained from DHI data (Matson et al., 2021). Cows were categorized as first-lactation or greater than first-lactation. Only data for first-lactation cows were used in the current study. Days for individual cows with no data on yield or milking, as well as no DIM and parity from the DHI records, were removed from the dataset. Days for individual cows where data were outside of 3 SD from the mean of the individual farm for that DIM were removed within farm. On average, there were 69 (range: 10 to 320) first-lactation cows per farm in the dataset, where cows had an average of 268 d of data (range: 41 to 305). A total of 4,217 cows were included in the dataset, which included a total of 1,131,670 days of data. Data were then summarized across all first-lactation cows on each farm for each DIM from 3 to 305 DIM. Variables included failures (no./d), milking frequency (no./d), refusals or rejections (no./d), visits (no./d), milk yield (kg/d), concentrate delivered (kg/d), visit duration per milking or box time (min/milking), milking interval (h), and SCC linear score.

Data were analyzed within certain DIM ranges to account for differences in stage of lactation and changes in AMS settings for the first-lactation cows. Graphing of the data for all farms for the first-lactation cows revealed a clear visual change in milking frequency and concentrate allocation at DIM 30, 60, 120, and 200 (Figure 1), likely due to the Lely AMS software settings related to milking permissions and concentrate allowance. Therefore, the DIM ranges chosen were 3 to 30, 31 to 60, 61 to 120, 121 to 200, and 201 to 305 DIM. The numerical month for each cow-day was averaged by DIM for each farm to account for time of year effects, including potential differences in climate and day length. This was also to account for the differing range of dates across the data collection period. Herd size was included in every model as a continuous variable to account for differing management styles between larger and smaller farms, as PRE farms were slightly smaller in general (Table 1). Herd size was not correlated with AMS stocking density, and therefore both variables were included in the models when applicable in order to account for management differences due to herd size, as well as potential behavior differences due to AMS stocking density.

**Figure 1:**
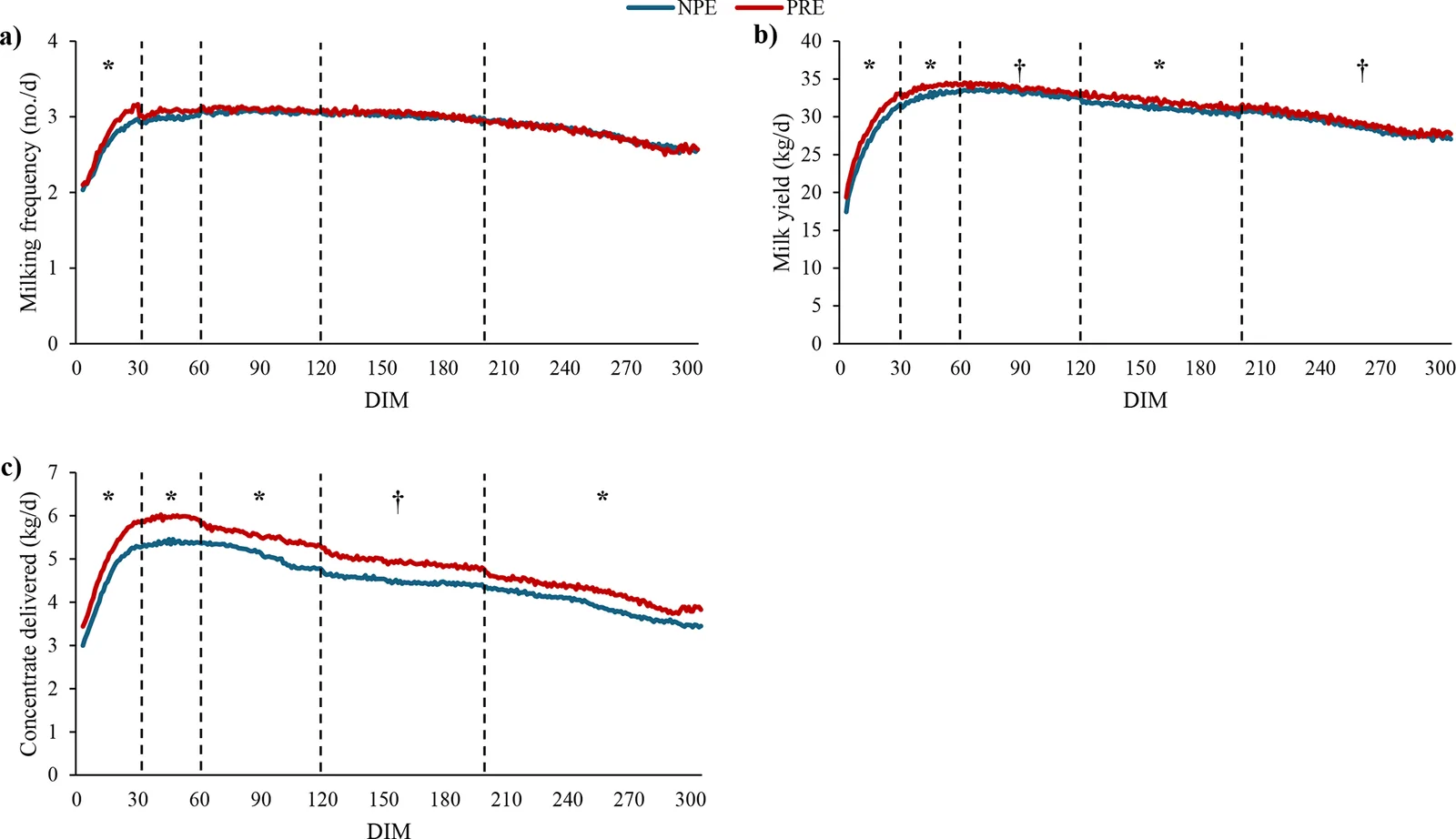
Least squares means of (a) milking frequency, (b) milk yield, and (c) concentrate delivered across the first lactation for farms that provided previous exposure to the automated milking system (AMS) before first calving (PRE; n = 27 farms) and farms that did not provide previous exposure (NPE n = 27 farms). * Indicates *P* ≤ 0.05 and † indicates 0.05 ≤ *P* < 0.10 between training categories for that DIM range (ranges indicated by vertical dashed lines).

All statistical analyses were performed using SAS 9.4 software (SAS Institute Inc., 2013). Farm was the experimental unit, and only data from the first-lactation cows within each farm were averaged and analyzed. All data were assessed for normality both visually and using the Shapiro–Wilk statistic (with *P* > 0.05 indicating normality) with the UNIVARIATE procedure of SAS. The outcome variables of milking frequency and visits were transformed using the natural log function, whereas the variables of rejections and milking interval were transformed using the square root function to achieve normality. Given non-normal distribution, the variable of failures was categorized to a binary variable (0 = less than or equal to an average of 0.1 failure events per day per cow for each DIM, and 1 = greater than an average of 0.1 failure events per day per cow for each DIM). The assumptions of normality were met for all other outcome data.

The association of previous AMS exposure and milking frequency, rejections, visits, milk yield, concentrate delivered, visit duration, milking interval, and linear score for first-lactation cows were analyzed using a linear regression analysis with the MIXED procedure of SAS with repeated measures (days) within each DIM range. The association of previous AMS exposure and odds of failures for first-lactation cows was analyzed using a logistic regression analyses with a binary distribution and LOGIT link using the GLIMMIX procedure of SAS with repeated measures (days) within each DIM range. Odds ratios for failures were determined using the ODDSRATIOS option. The models for both the MIXED and GLIMMIX procedures included the fixed effects of previous exposure category (NPE or PRE), DIM, and herd size. Pair was included as a random effect. To model repeated measures across time using DIM as the repeated measure, with farm within pair as the subject of the repeated statement, the variance-covariance matrix structure used was autoregressive or compound symmetry. The variance-covariance matrix structures were chosen on the basis of best fit according to Schwarz's Bayesian information criterion for the MIXED procedure, or the generalized chi-squared to degrees of freedom ratio for the GLIMMIX procedure. Degrees of freedom for fixed effects were estimated using the Kenward–Roger option in the MODEL statement. Several covariates were tested and retained in the models when *P* ≤ 0.05. Covariates (see Table 1) retained for outcome variables were as follows: milking frequency (AMS stocking density, average month of the year for each DIM, access to outside), rejections (AMS stocking density, average month of the year for each DIM, ventilation type, frequency of stall raking per day), visits (AMS stocking density, average month of the year for each DIM), yield (average month of the year for each DIM, years since AMS was installed, ventilation type, access to outside), concentrate delivered (average month of the year for each DIM, was a tiestall system before converting to AMS), visit duration (average month of the year for each DIM), milking interval (AMS stocking density, average month of the year for each DIM, ventilation type, access to outside), linear score (average month of the year for each DIM, frequency of stall raking per day, alley cleaning frequency, frequency of bedding added), and failures (AMS stocking density, average month of the year for each DIM, and years since AMS was installed). Interactions between DIM and training category were explored. However, no interactions were detected (*P* > 0.05) and thus are not further reported.

Previous exposure to the AMS before first calving was associated with greater milking frequency (Figure 1a; Table 2), shorter milking interval (Table 2), and 1.32× lower odds of having an average of 0.1 or more failures per day per cow (odds ratio [**OR**] = 0.76; 95% CI = 0.60–0.97; *P* = 0.02) across the first 30 DIM. Greater milking frequency, shorter milking interval, and more successful milkings likely contributed to the increased milk yield across the first 30 DIM for PRE farms (Figure 1b; Table 2). Greater milk yield has been observed in cows previously trained to the AMS (Brasier et al., 2025a,b) and, in this case, may be related to the enhanced proliferation of secretory tissue in the mammary gland due to a shorter milking interval (Weiss et al., 2005; Penry et al., 2018) and the associated greater milking frequency (Hale et al., 2003). Given that AMS settings and fetching records could not be obtained, we cannot conclude if previous exposure and improved adaptation were the only cause for the increased milking frequency and shorter milking intervals for PRE farms. It is possible that PRE farms had shorter milking permissions for only the first 30 DIM compared with NRE farms, or PRE farms fetched cows more often per day. However, AMS visits and rejections were numerically higher for PRE farms during the first 30 DIM and from 31 to 60 DIM (Table 2), which might provide additional evidence that first-lactation cows on PRE farms adapted better to the AMS. Researchers have demonstrated that milking interval will influence yield (Penry et al., 2018) and behavior (Schwanke et al., 2025); however, limited research is available on the influence of different milking permissions. Therefore, future research should be focused on the influence of milking permission on behavior and milk yield in AMS.

**Table 2:** Association between previous exposure to the automated milking system (AMS) before first calving on Canadian AMS farms and AMS milking performance across multiple DIM ranges for the first-lactation cows

Variable^2^	Category^1^		95% CI		*P*-value
	NPE	PRE	NPE	PRE	
3 to 30 DIM					
Milking frequency^3^ (no./d)	2.6	2.7	2.59–2.62	2.67–2.75	0.04
Rejections^3^ (no./d)	1.4	1.5	1.17–1.29	1.46–1.63	0.33
Visits^3^ (no./d)	4.1	4.3	3.83–4.01	4.29–4.53	0.17
Milk yield (kg/d)	26.8	28.6	26.70–27.43	28.06–28.73	<0.001
Concentrate delivered (kg/d)	4.4	5.0	4.37–4.52	4.86–5.04	0.02
Visit duration (min/milking)	7.2	7.1	7.17–7.26	7.04–7.14	0.29
Milking interval^3^ (h)	10.0	9.5	9.81–10.01	9.31–9.55	0.04
Linear score^4^	3.0	2.8	2.99–3.09	2.75–2.84	0.04
31 to 60 DIM					
Milking frequency^3^ (no./d)	3.0	3.1	2.98–3.03	3.14–3.19	0.19
Rejections^3^ (no./d)	1.6	1.8	1.50–1.61	1.75–1.92	0.34
Visits^3^ (no./d)	4.7	4.9	4.68–4.82	4.80–5.01	0.35
Milk yield (kg/d)	32.7	33.8	32.56–32.98	33.70–34.08	0.005
Concentrate delivered (kg/d)	5.4	5.9	5.35–5.46	5.89–6.02	0.008
Visit duration (min/milking)	7.7	7.5	7.61–7.71	7.44–7.52	0.24
Milking interval^3^ (h)	8.5	8.3	8.45–8.59	8.20–8.32	0.36
Linear score^4^	2.5	2.4	2.48–2.56	2.32–2.41	0.05
61 to 120 DIM					
Milking frequency^3^ (no./d)	3.1	3.1	3.09–3.12	3.13–3.17	0.66
Rejections^3^ (no./d)	1.6	1.7	1.54–1.62	1.73–1.83	0.75
Visits^3^ (no./d)	4.7	4.8	4.64–4.74	4.77–4.81	0.78
Milk yield (kg/d)	33.1	33.8	32.94–33.23	33.71–34.02	0.07
Concentrate delivered (kg/d)	5.1	5.5	5.04–5.11	5.49–5.59	0.02
Visit duration (min/milking)	7.3	7.1	7.26–7.32	7.07–7.13	0.21
Milking interval^3^ (h)	8.4	8.3	8.38–8.47	8.21–8.30	0.37
Linear score^4^	2.6	2.4	2.56–2.62	2.42–2.48	0.03
121 to 200 DIM					
Milking frequency^3^ (no./d)	3.0	3.0	3.03–3.07	3.06–3.09	0.73
Rejections^3^ (no./d)	1.8	1.8	1.73–1.84	1.78–1.87	0.95
Visits^3^ (no./d)	4.7	4.7	4.67–4.80	4.75–4.86	0.92
Milk yield (kg/d)	31.2	32.0	31.02–31.31	31.99–32.27	0.001
Concentrate delivered (kg/d)	4.5	4.9	4.49–4.60	4.89–4.98	0.08
Visit duration (min/milking)	6.8	6.7	6.79–6.85	6.68–6.73	0.29
Milking interval^3^ (h)	8.5	8.4	8.48–8.56	8.39–8.47	0.72
Linear score^4^	2.7	2.5	2.70–2.75	2.44–2.50	0.01
201 to 305 DIM					
Milking frequency^3^ (no./d)	2.8	2.8	2.81–2.84	2.79–2.82	0.88
Rejections^3^ (no./d)	1.8	1.8	1.79–1.86	1.76–1.83	0.95
Visits^3^ (no./d)	4.6	4.5	4.62–4.70	4.55–4.65	0.93
Milk yield (kg/d)	28.9	29.4	28.72–29.18	29.22–29.46	0.08
Concentrate delivered (kg/d)	3.9	4.2	3.85–3.91	4.20–4.28	0.04
Visit duration (min/milking)	6.6	6.5	6.57–6.62	6.49–6.53	0.60
Milking interval^3^ (h)	9.3	9.3	9.36–9.44	9.30–9.38	0.92
Linear score^4^	2.7	2.7	2.74–2.78	2.69–2.74	0.56

1 NPE = no previous exposure to the AMS before first milking (n = 27 farms); PRE = had previous exposure to the AM before first milking (n = 27 farms).

2 Farm average of data collected for first-lactation cows across the DIM period.

3 Back transformed.

4 Linear score calculated as LS = [log2(SCC/100,000)] + 3. Four PRE farms and 5 NPE farms did not collect SCC data.

The lower odds of milking failures for PRE farms during the first 30 DIM may be due to less aversive reactions during milking (Neupane et al., 2026), as cows may have been habituated to the AMS during training (Brasier et al., 2025a,b). This may include fewer kickoff events or less movement during attachment for cows that experienced previous exposure to the AMS (Brasier et al., 2025a,b), resulting in more successful milkings for PRE farms (Neupane et al., 2026). More successful milkings and greater milk yield for PRE farms may also be associated with the lower SCC linear score for PRE farms during the first 30 DIM (Table 2). Researchers have demonstrated that training can result in lower milk conductivity (von Kuhlberg et al., 2021) and a lower SCC (Whitehead, 2024), suggesting better udder health for trained cows. Fewer failures for PRE farms may be associated with the reduced SCC linear score, as Neupane et al. (2026) demonstrated that cows diagnosed with mastitis had a higher odds of a failure event compared with healthy cows. Greater milk yield may also have contributed to the lower SCC linear score for PRE farms due to greater milk dilution (Green et al., 2006).

Milk yield was either greater, or tended to be greater, for PRE farms at different stages across the entire first lactation (Figure 1b; Table 2), despite no sustained differences in milking frequency (Figure 1a; Table 2) or interval (Table 2) for the remainder of the first lactation. Therefore, the improved milk yield could be a carryover effect of the improved adaptation in the first 30 DIM, as a greater milking frequency in early lactation is known to have positive effects on lactational performance and persistency (Hale et al., 2003; Pettersson et al., 2011), resulting in higher mean milk revenue (Beaudry et al., 2026). Increased milk yield across the lactation was potentially also related to fewer milking failures and lower SCC linear score. The PRE farms had 1.32× lower odds from 31 to 60 DIM (OR = 0.76; 95% CI = 0.60–0.97; *P* = 0.03) and 1.56× lower odds from 121 to 200 DIM (OR = 0.64; 95% CI = 0.53–0.77; *P* < 0.001) of having an average of 0.1 or more failures per day per cow compared with NPE farms. No differences were detected from 61 to 120 DIM or from 201 to 305 DIM for failure events (*P* > 0.1). Similar to that discussed for the first 30 DIM, fewer failures for PRE farms may have contributed to increased milk yield across the lactation, as well as the lower SCC linear scores from 3 to 200 DIM for the PRE farms (Table 2). Additionally, persistent greater milk yield could be due to better udder health for PRE farms (Hand et al., 2012), but that greater milk yield could also be diluting the milk and thus lowering the SCC linear score (Green et al., 2006).

The PRE farms were also associated with having greater, or a tendency for greater, AMS concentrate delivered at different stages of lactation (Figure 1c; Table 2). The concentrate allocated in the AMS is typically based on stage of lactation and milk yield (Bach and Cabrera, 2017); therefore, PRE farms likely allocated more concentrate compared with NPE farms due to having greater milk yield. Increased energy intake from greater concentrate provided at the AMS may have contributed to the higher milk yield for PRE farms (Schwanke et al., 2024); however, providing more AMS concentrate does not always result in greater yield due to cows balancing their intake of AMS concentrate and PMR, often resulting in similar total DMI (Bach et al., 2007; Schwanke et al., 2025). The nutrient density of both the concentrate and PMR could influence milking behavior and yield. However, in an analysis from a portion of the total population of farms in this study, limited associations were detected between nutrient density of both the PMR and concentrate and milking behavior and yield (Van Soest et al., 2024). Although diets fed were different between herds (Van Soest et al., 2024), pair matching based on major forage and region should have accounted for some of those nutrient differences. It is also important to note that there may be concerns with feeding high amounts of AMS concentrate. As discussed by Bach and Cabrera (2017), cows do not always receive or consume all of the concentrate delivered if allocation is too high, which can cause a large variation in nutrient supply; this can in turn result in negative effects on milk production.

Due to the small number of farms that used previous exposure in the current dataset, the PRE farms could not be separated into different methods and durations of previous exposure. This is a limitation, as the type of training can influence adaptation and production (Brasier et al., 2025b). Another major limitation of this study was that milking permissions and AMS concentrate settings were not known for any of the farms, which would have an influence on milking frequency, interval, rejections, milk yield, and concentrate delivered (Schwanke et al., 2025). Other differences between herds that were not able to be obtained may also have influenced results, such as genetics and other management factors not accounted for.

In conclusion, farms that exposed first-lactation cows to the AMS before first milking were associated with a greater milking frequency and a shorter milking interval across the first 30 DIM, indicating improved adaptation to the AMS. This previous exposure to the AMS was also associated with either greater milk yield, or a tendency for greater milk yield, at different stages of lactation across 305 DIM, as well as a lower SCC linear score up to 200 DIM, and lower odds of milking failures from 3 to 60 and 120 to 200 DIM. This indicates that the improved adaptation due to previous exposure may not only improve performance in early lactation, but may also have persistent effects through the first lactation. These results complement the growing body of evidence demonstrating the benefits of some type of training through previous exposure before first milking on the AMS.
